# Red Algae “*Sarcodia suieae*” Acetyl-Xylogalactan Downregulate Heat-Induced Macrophage Stress Factors Ddit3 and Hyou1 Compared to the Aquatic Animal Model of Nile Tilapia (*Oreochromis niloticus*) Brain Arachidonic Acid Expression

**DOI:** 10.3390/ijms232314662

**Published:** 2022-11-24

**Authors:** Po-Kai Pan, Kuang-Teng Wang, Fan-Hua Nan, Tsung-Meng Wu, Yu-Sheng Wu

**Affiliations:** 1 Department of Aquaculture, National Pingtung University of Science and Technology, Pingtung 912, Taiwan; 2 Department of Aquaculture, National Taiwan Ocean University, Keelung 202, Taiwan

**Keywords:** *Sarcodia suieae*, acetyl-xylogalactan, environment, stress, apoptosis

## Abstract

Anthropogenic climate change is known to be an increased stress that affects aquatic animal behavior and physiological alternations, which can induce the animal’s death. In order to known whether the extracted acetyl-xylogalactan function on the regulation of the external high temperature induced death, we first selected the mammalian cell line “RAW 264.7” used in the previous experiment to evaluate the extracted acetyl-xylogalactan function. We aimed to evaluate the effects of the acetyl-xylogalactan on the RAW 264.7 macrophages and Nile Tilapia stress factor expression under the heat environment. In the in vitro cell observation, we assessed the cell survival, phagocytic activity, intracellular Ca^2+^ level, mitochondria potential exchange, apoptotic assay findings, galactosidase activity, RNA-seq by NGS and real-time polymerase chain reaction (QPCR) expression. In the in vivo Nile Tilapia observation aimed to evaluate the blood biochemical indicator, brain metabolites exchange and the liver morphology. In our evaluation of RAW 264.7 macrophages, the RNA sequencing and real-time polymerase chain reaction (PCR) was shown to upregulate the expression of the anti-apoptosis *Cflar* gene and downregulate the expression of the apoptosis factors *Ddit3* and *Hyou1* to protect macrophages under heat stress. We already knew the extracted acetyl-xylogalactan function on the mammalian “RAW 264.7” system. Following, we used the aquatic Nile Tilapia model as the anthropogenic climate change high temperature experiment. After feeding the Nile Tilapia with the acetyl-xylogalactan, it was found to reduce the brain arachidonic acid (AA) production, which is related to the NF-κB-induced apoptosis mechanism. Combined with the in vitro and in vivo findings, the acetyl-xylogalactan was able to reduce the heat induced cell or tissue stress.

## 1. Introduction

Previous studies reported that polysaccharides extracted from *Laminaria japonica* and seaweed had a positive function on the anti-tumour, immune cytokine-regulatory, anti-apoptosis and anti-inflammatory activity [1,2] and were even able to protect the host against the infection. With currently rising CO_2_ levels in atmosphere and marine surface waters as well as the projected scenarios of CO_2_ disposal in the ocean, it has been emphasized that CO_2_ sensitivities need to be investigated in aquatic organisms, especially in animals which may well be the most sensitive [3]. Further, it is already known that internal high temperature is a major physiological stress, resulting in the clinical manifestation of chronic and acute illnesses [4]. Additionally, the evidence illustrated that the fever and hyper-inflammation indicates to increase the body temperature, the major clinical febrile phenomenon [5].

In the fever and hyper-inflammatory responses, interactions of the immune cellular and microenvironments can minimize impending injury or infection and restore tissue homeostasis [6]. However, an un-controlled inflammation response may lead to a chronic inflammatory disease [7], for example, the increased permeability of the vascular endothelium, allowing the leakage of serum components and extravasation of immune cells [8]. With the un-controlled febrile, it enhances the activity of reactive oxygen species (ROS), resulting in damage to cellular proteins and DNA [9]. This phenomenon is known as heat stress. Heat stress in the cellular response is known to reduce alterations in host physiological and immune systems, which are associated with cellular death, impaired growth [10] and enhanced mucosal damage [11]. Heat stress is known to lead to cell apoptosis through the multiple forms of DNA damage, such as base damage, single-strand breaks, replication inhibition and double-strand breaks [12,13,14]. Research already shows that heat stress enhances cell apoptosis via the phosphorylation of the eIF2α [15].

Human clinical therapy have also been reported to regulate pro-apoptotic genes through NF-kappa B [16]. The use of the pyrin was able to reduce the macrophages apoptosis from the pyrin-truncated mice through an IL-1-independent pathway [17]. In our previous research, we found the acetyl-xylogalactan extracted from *Sarcodia suieae* function in the regulation of the NF-Kappa B pathway to produce the IL-1 beta [18]. Combined with these previous findings, we thought that the acetyl-xylogalactan may reduce macrophage apoptosis similarly to the pyrin mechanism. For reference, the intracellular Ca^2+^ homeostasis was related to cell death through the action of the proteolytic enzymes. Additionally, the intracellular targets of apoptotic Ca^2+^ signal was with the special emphasis of the mitochondria on the cytosolic Ca^2+^-dependent enzymes [19,20].

In this study, we used the RAW 264.7 to evaluate the function of the acetyl-xylogalactan on the heat-induced RAW 264.7 RNA-seq and real-time PCR stress factor expression. The evidence was shown to positively downregulate apoptotic factors. Furthermore, we aimed to evaluate the effects of *Sarcodia suieae* acetyl-xylogalactan on the Nile Tilapia stress response under a high temperature environment.

## 2. Results

### 2.1. Cellular Exeperiment

#### 2.1.1. RAW 264.7 Macrophage Survival and Phagocytotic Activity under Heat Stress

**Figure 1:**
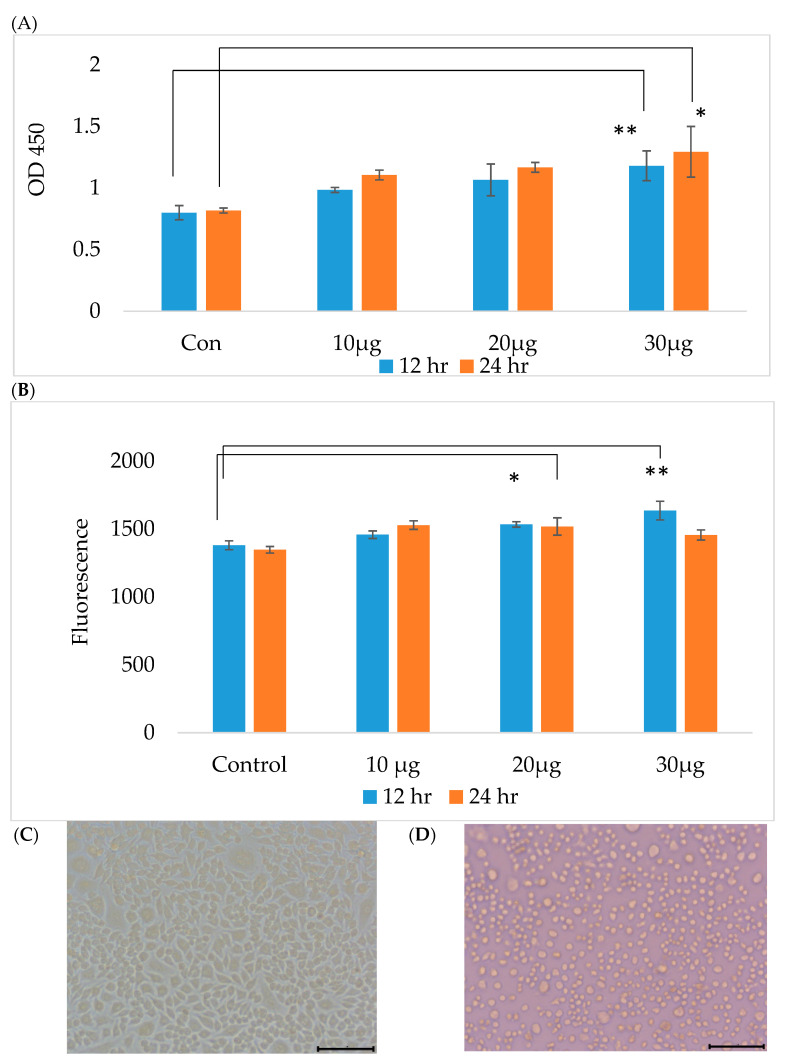
RAW 264.7 macrophage survival and phagocytotic activity of: (**A**) The 30 μg/mL treatment group showed significantly greater survival than the control group (*p* < 0.01) at 12 and 24 h (*p* < 0.05) under heat stress; (**B**) acetyl-xylogalactan treatment was able to significantly induce phagocytic ability (*p* < 0.05) at 12 h under heat stress, but not at 24 h; (**C**) the RAW 264.7 cells displayed an oval shape at 37 °C; (**D**) The RAW 264.7 cells showed at 41 °C (the black bar was shown as 50 μm) (*p* < 0.05 was marked as *, *p* < 0.01 was marked as **).

Based on these findings, we chose a heat stress temperature of 41 °C. In the cell survival experiment, RAW 264.7 macrophages were treated with 10, 20 and 30 μg/mL acetyl-xylogalactan for 12 and 24 h at 41 °C. The survival of the treatment groups at 41 °C was greater than those in the control group. In this analysis, the 20 and 30 μg/mL treatment groups showed significantly greater survival than the control group (*p* < 0.05) at 12 h, as shown in Figure 1A. At 24 h, the 30 μg/mL treatment group showed significantly greater survival than the control group (*p* < 0.05). Based on these findings, we hypothesized that the Sarcodia suieae acetyl-xylogalactan was able to maintain RAW 264.7 macrophage survival under heat stress. The results presented in Figure 1B show that acetyl-xylogalactan treatment was able to significantly induce phagocytic ability (*p* < 0.05) at 12 h under heat stress. However, at 24 h, the phagocytic ability did not differ between groups (*p* > 0.05). Thus, RAW 264.7 macrophages treated with acetyl-xylogalactan were able to maintain their phagocytic ability under heat stress for 12 h. The cell phenomenon of the RAW 264.7 under the 37 and 41 °C was presented as Figure 1C,D. It should be noted that at a normal temperature (37 °C), the RAW 264.7 was shown as having an oval shape when compared to the high temperature conditions (41 °C).

#### 2.1.2. RAW 264.7 Macrophage Apoptosis and Galactosidase Observation under Heat Stress

**Figure 2:**
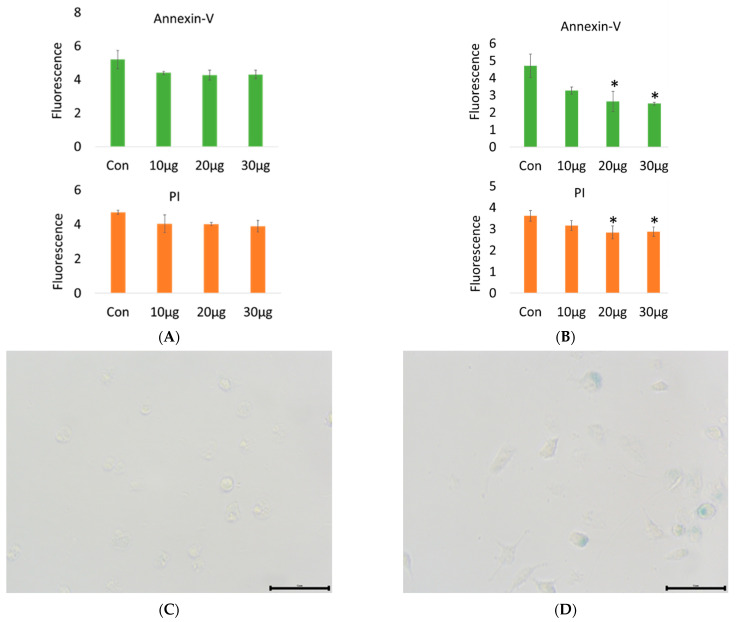
RAW 264.7 macrophage apoptosis detection via Annexin-V: (**A**) Treatment with 20 µg/mL acetyl-xylogalactan was able to significantly reduce apoptosis (*p* < 0.05) at 12 h under heat stress. (**B**) However, apoptosis did not show significant differences among groups at 24 h (*p* > 0.05). (**C**) RAW 264.7 macrophage galactosidase detection at a normal temperature (37 °C). (**D**) 30 μg/mL acetyl-xylogalactan significantly induced galactosidase production (blue-stained cells) at 41 °C (the black bar was shown as 50 μm) (*p* < 0.05 was marked as *).

The results in Figure 2A show that 20 µg/mL acetyl-xylogalactan treatment was able to significantly reduce apoptosis (*p* < 0.05) at 12 h under heat stress. At 24 h under heat stress, apoptosis did not show significant differences among the groups (*p* > 0.05), as shown in Figure 2B. According to our findings, we observed that the acetyl-xylogalactan was able to reduce the Annexi-V and PI rate at 12 hr. However, at the 24 h point, we did not see the significant Annexin-V and PI rate reduction compared to the control.

In the Figure 2C, the normal temperature (37 °C) was not induced the RAW 264.7 galactosidase production. The findings in Figure 2D show that treatment with 30 μg/mL acetyl-xylogalactan was able to significantly induce galactosidase production (blue-stained cells) at 12 h under 41 °C heat stress. In contrast, galactosidase production was not observed in the control group. We proposed that under 41 °C, the RAW 264.7 macrophage without treatment already displayed a programmed death. While treated with acetyl-xylogalactan, it was able to prevent the direct death cell under 41 °C. Combined with the the RNA-seq analysis, it was also known to display non-significant lactate dehydrogenase A expression after treatment for 24 h [18].

#### 2.1.3. Intracellular Ca^2+^ Concentration and Mitochondrial Membrane Potential of the Pre-Treated RAW 264.7 after Heat Stress

**Figure 3:**
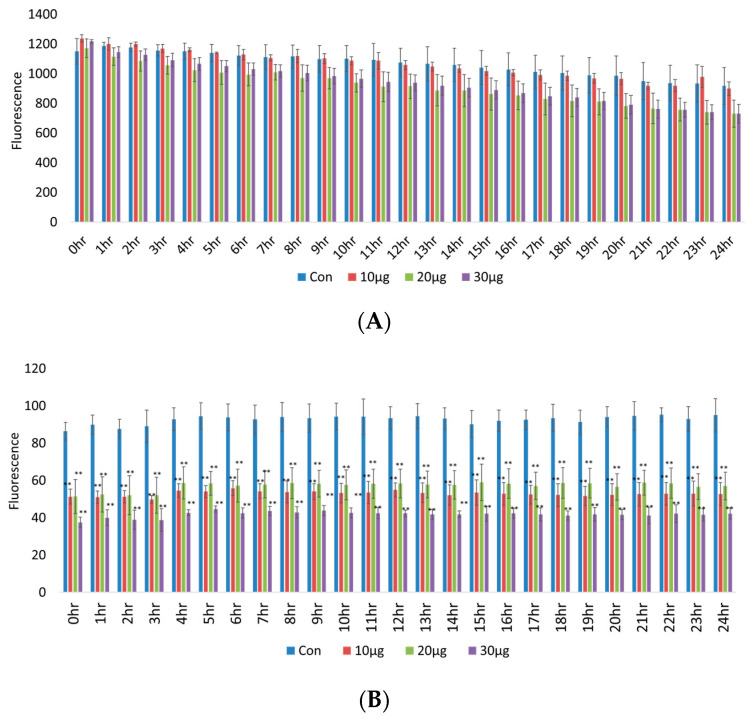
RAW 264.7 macrophage intracellular Ca^2+^ concentration and mitochondria membrane potential: (**A**) There was no significant change (*p* > 0.05) under pretreatment with various concentrations of acetyl-xylogalactan following incubation at 41 °C. (**B**) Mitochondrial membrane potential recovery was lower in the treatment groups than in the control group (*p* < 0.01) (Treatment was presented as 10 μg means the 10 μg acetyl-xylogalactan/mL (culture medium); 20 μg means the 20 μg acetyl-xylogalactan/mL (culture medium); 30 μg means the 30 μg acetyl-xylogalactan/mL (culture medium)) (*p* < 0.01 was marked as **).

RAW 264.7 intracellular Ca^2+^ concentration recovery is presented in Figure 3A, which shows that there was no significant change (*p* > 0.05) under pretreatment with various concentrations of acetyl-xylogalactan for 24 h following incubation under heat stress. However, the macrophage mitochondrial membrane potential recovery in the treatment groups was lower than in the control group (*p* < 0.01), as shown in Figure 3B.

Thus, treatment with acetyl-xylogalactan increased the RAW 264.7 mitochondrial membrane potential recovery to reduce intracellular stress. We hypothesized that acetyl-xylogalactan was able to regulate the macrophage intracellular stress induced by the elevated temperature.

#### 2.1.4. RNA Sequencing (Transcriptome)

**Figure 4:**
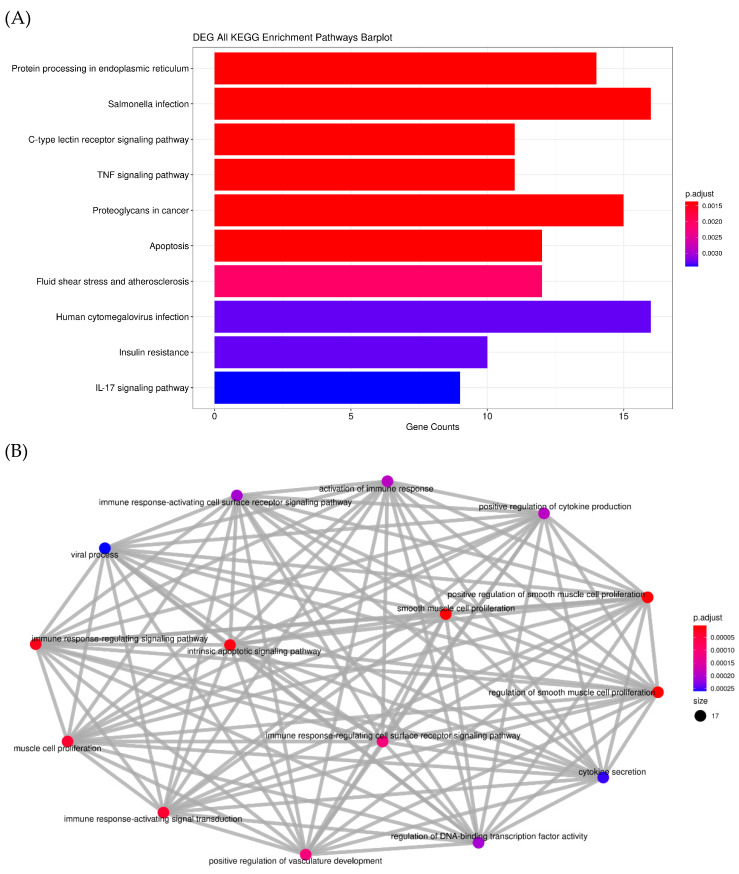

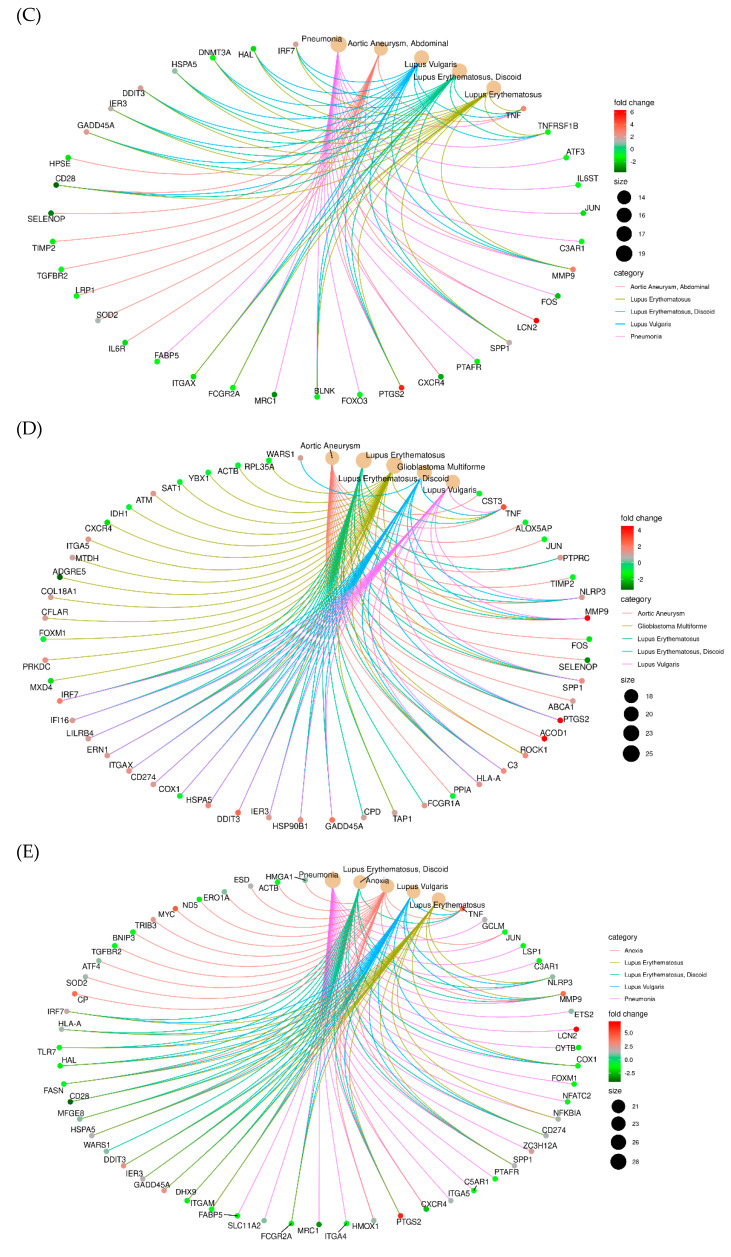

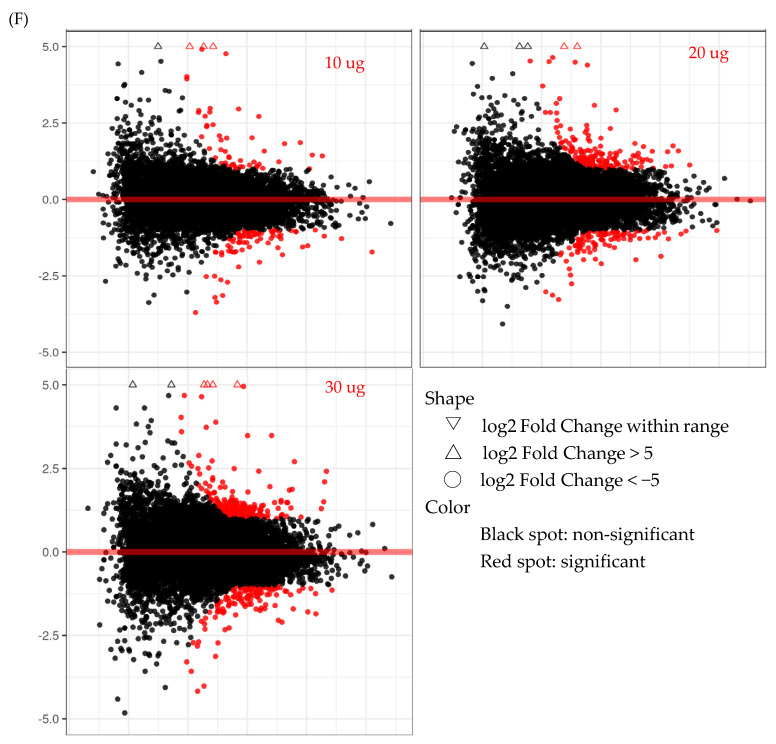
RNA sequencing (transcriptome) analysis: (**A**) Acetyl-xylogalactan may have affected the counts of the apoptosis gene, as 10 counts were presented in the KEGG analysis. (**B**) Acetyl-xylogalactan may have regulated the relative cell phenomenon; (**C**) log two-fold change of the acetyl-xylogalactan various concentration treatment. Totals of 10 μg (**D**), 20 μg (**E**), 30 μg (**F**) Volcano map of the treatments with various concentrations of acetyl-xylogalactan treatment. (Treatment was presented; 10 μg means 10 μg of acetyl-xylogalactan/mL (culture medium); 20 μg means 20 μg of acetyl-xylogalactan/mL (culture medium); 30 μg means 30 μg of acetyl-xylogalactan/mL (culture medium)).

Macrophage RNA was isolated and mapped to the reference genome. RNA-seq analysis showed that RNA and gene expression significantly changed with ace-tyl-xylogalactan treatment. After the sequencing was completed using the predicted pathway of DEG analysis, the acetyl-xylogalactan may have affected the counts of the apoptosis gene, as 10 counts were presented in the KEGG and GO term analysis, as shown in Figure 4A,B. DEG analysis demonstrates that the acetyl-xylogalactan was able to regulate the RAW 264.7 intrinsic apoptosis phenomenon Figure 4A. In Figure 4A,B, the gene ontology (GO) analysis also demonstrated that treatment with acetyl-xylogalactan was able to regulate the RAW 264.7 biological process (BP) target.

After the log two-fold change analysis of the NGS, we observed that the 10 μg/mL, 20 μg/mL and 30 μg/mL treatment groups were able to regulate the significant difference in gene expression, as shown in Figure 4B. Treated with 10 μg/mL (Figure 4C), 20 μg (Figure 4D) and 30 μg (Figure 4E) of acetyl-xylogalactan, the RAW 264.7 was enhanced in the different genes, such as the DDIT3, GADD45A etc. By the results in Figure 4F, we can see that the enhanced gene increased with the treated concentration. 

**Figure 5:**
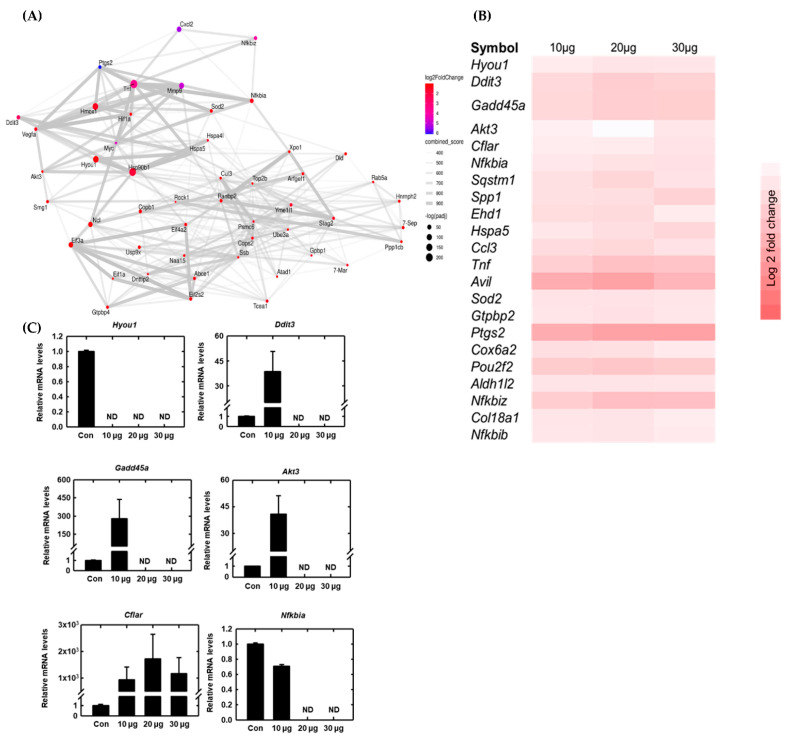
The RNA- seq analysis: (**A**) The network of the relative RNA gene; (**B**) the heatmap of the RNA-seq analysis, presented with a log two-fold change; (**C**) mRNA expression. Acetyl-xylogalactan downregulated apoptosis under heat stress via target genes, such as Nfkbia, Ddit3, Gadd45a, Akt3 and Hyou1, and upregulated Cflar expression. (Treatment was presented; 10 μg means 10 μg of acetyl-xylogalactan/mL (culture medium); 20 μg means 20 μg of acetyl-xylogalactan/mL (culture medium); 30 μg means 30 μg of acetyl-xylogalactan/mL (culture medium)).

According to the DEG and GO summarize, we understand the potential role of the acetyl-xylogalactan to the RAW 264.7 under heat stress. Thereafter, the proposed genes Nfkbia, Ddit3, Gadd45a, Akt3, Hyou1 and Cflar were examined. The results showed that acetyl-xylogalactan downregulated apoptosis under heat stress via target genes, such as Nfkbia, Ddit3, Gadd45a, Akt3 and Hyou1 Figure 5A,B.

However, acetyl-xylogalactan was shown to upregulate the expression of Cflar. Taken together, these findings indicate that the *S. suieae* acetyl-xylogalactan treatment reduced the expression of apoptosis factors under heat stress, as shown in Figure 5C.

#### 2.1.5. Cytokines IL-6 and IL-17 A Production

**Figure 6:**
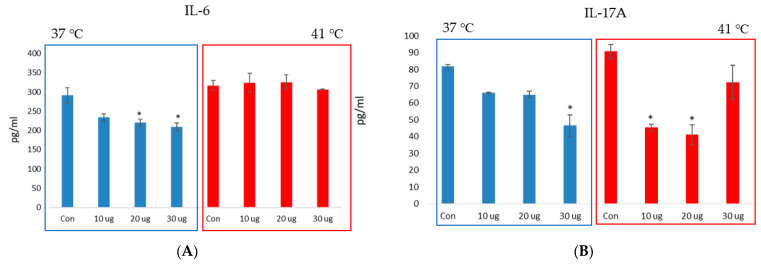
Effect of the RAW 264.7 macrophage cytokine IL-6 and IL-17A production of: (**A**) the blue bar means the RAW 264.7 IL-6 production at 37 °C; red bar means the RAW 264.7 IL-6 production at 41 °C. (**B**) the blue bar means the RAW 264.7 IL-17A production at 37 °C; red bar means the RAW 264.7 IL-17A production at 41 °C. (* Treatment was presented; 10 μg means 10c of μg acetyl-xylogalactan/mL (culture medium); 20 μg means 20 μg of acetyl-xylogalactan/mL (culture medium); 30 μg means 30 μg of acetyl-xylogalactan/mL (culture medium)) (*p* < 0.05 was marked as *).

IL-6 and IL-17A production in RAW 264.7 macrophages treated with or without *S. suieae* acetyl-xylogalactan was analysed under heat stress. In brief, 1 × 10^6^ RAW 264.7 macrophages were cultured in a 96-well plate with or without 10, 20 or 30 μg/mL *S. suieae* acetyl-xylogalactan at 37 and 41 °C for 24 h. The culture medium was then analysed using the ELISA IL-6 and IL-17A assay kit (QIAGEM, SEM03015A and SEM03023A). The analysis data are shown in Figure 6, according to the IL-6 results, and at 37 °C, the acetyl-xylogalactan was able to reduce the RAW 264.7 IL-6 production (Figure 6A, blue bar; *p* < 0.05). However, at 41 °C, the IL-6 was not significantly different between control and treatment (Figure 6A, red bar; (*p* > 0.05)).

Moreover, for IL-17A, at 37 °C, the acetyl-xylogalactan was able to reduce the RAW 264.7 IL-17A production at 30 μg/mL (Figure 6B, blue bar; *p* < 0.05). However, at 41 °C, the IL-17A was able to significantly reduce IL- 17A production at 10 and 20 μg/mL (Figure 6B, red bar; (*p* < 0.05)).

### 2.2. In Vivo Animal Expeiment

#### 2.2.1. Blood Biochemical Analysis

Hematologic and blood biochemical parameters reflect alterations in physiological responses as shown in Table 1.

Feeding with 0 g/Kg-Feed (control feed), 10 g/Kg-Feed, 20 g/Kg-Feed or 30 g/Kg-Feed acetyl-xylogalactan under 35 °C compared to the 25 °C control treatment. The T-Bil level was significantly higher in the 10 g/Kg-Feed group at 35 °C than the 25 °C control group (*p* < 0.05).

Feeding with 0 g/Kg-Feed (control feed), 10 g/Kg-Feed, 20 g/Kg-Feed, or 30 g/Kg-Feed acetyl-xylogalactan under 35 °C compared to the 25 °C control treatment. The experiment was significantly lower in the 0 g/Kg-Feed (control feed) and 10 g/Kg-Feed group at 35 °C than the 25 °C control group (*p* < 0.05).

However, regarding the Glu, T-Cho, BUN, GOT, GPT, T-Pro, Alb, Ca, TG, UA, LDH, Alb/T-Pro, CPK, ALP, Mg, HDL-c, IP, GGT, Cre, FRA and IL-10 levels no significant differences were observed between different groups (*p* > 0.05).

#### 2.2.2. Brain Metabolites Analysis

**Figure 7:**
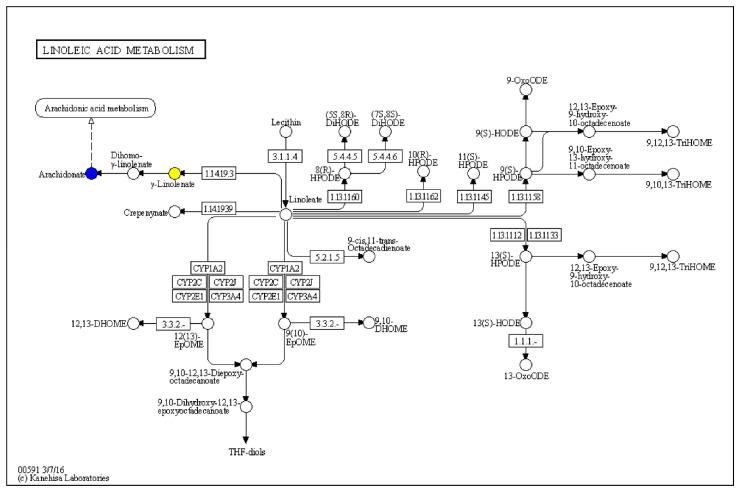
The KEGG analysis of the metabolites pathway. The blue mark denotes *p* value <0.05. The yellow mark denotes *p* value > 0.1.

The metabolites analysis was shown as Table 2. A comparison of the 35 °C—20 g/Kg-Feed, and 35 °C—30 g/Kg-Feed acetyl-xylogalactan compared to the 25 °C metabolites, revealed that “Arachidonic acid” presented with a lower expression (35 °C—20 g/Kg-Feed vs. 25 °C, *p*-value = 0.018, log2 fold change = −0.88, and 35 °C—30 g/Kg-Feed vs. 25 °C, *p*-value = 0.04, log2 fold change = −0.95). “Ginkgolide A” was presented with a downregulation at 35 °C—0, 10, 20 and 30 g/Kg-Feed compared to the 25 °C. As the KEGG analysis as Figure 7, the metabolites were revealed to affect the “Linoleic acid metabolism”.

#### 2.2.3. Histological Observation

**Figure 8:**
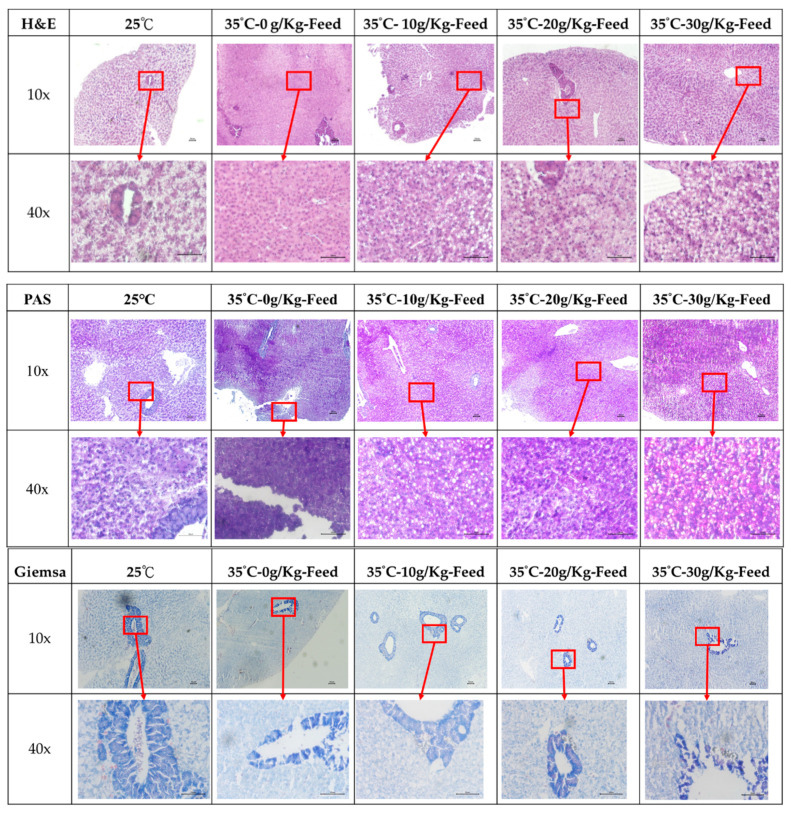
Histological observation of the liver stained by the H&E, PAS and Giemsa stain. The black bar was presented as 50 μm.

As shown in Figure 8, 25 °C presented a normal liver tissue structure and morphology, while heat stress (35 °C) shows hepatic steatosis (blue arrow) by the H&E stain at each feeding acetyl-xylogalactan groups. The observation of the hepatic glycogen are shown in Figure 8. Compared with the control (25 °C), it significantly increased the hepatic glycogen (orange arrow) observation at the 35 °C–30 g/Kg-Feed acetyl-xylogalactan by the PAS stain.

## 3. Discussion

Heat stress (HS) is known to induce a significant increase in cytosolic Ca^2+^ concentration. Further evidence demonstrates that Ca^2+^ might be a factor in the regulation of ganoderic acid (GA) biosynthesis and the accumulation of heat shock proteins (HSP) production [21]. A previous study evaluated the response of a chicken macrophage-like (HD11) cell line to heat stress and LPS stimulation and has showed that the monocyte/macrophage cell line responded to heat stress with an increased mRNA abundance of the HSP [22]. One study revealed that heat stress (HS) induced the apoptosis of the vascular endothelial cells associated with reactive oxygen species (ROS)-induced p53 translocation into the mitochondria to induce changes in intracellular Ca^2+^ levels [23]. Intracellular Ca^2+^ level through cation channels, mitochondrial function and oxidative stress-induced parallel pathophysiological mechanisms in diabetic neuropathy. Impaired insulin signaling and Ca^2+^ influx through transient receptor potential (TRP) channel and voltage-gated Ca^2+^ channel activations triggers sensory neuron mitochondrial depolarization [24].

In our study, we found that acetyl-xylogalactan was not significantly affected in the macrophage recover intracellular Ca^2+^ after heat stress. However, our assessment of mitochondrial membrane potential indicated the recovery of membrane potential after treatment with acetyl-xylogalactan under heat stress. This phenomenon was similar to the intracellular Ca^2+^ expression and apoptotic genes downregulation. We propose that treatment with acetyl-xylogalactan is able to inhibit intracellular stress to reduce the mitochondrial-mediated damage signaling leading to cell death.

In our previous study, we examined whether acetyl-xylogalactan affected the macrophage phagocytic ability without heat stress at 37 °C. Our published results showed that a high concentration of 30 μg/mL was able to increase the phagocytic ability during 12 h treatment. However, phagocytic ability was not enhanced after the 24 h treatment [18]. Based on our findings in this research, we observed the phagocytic ability exchange under heat stress with or without acetyl-xylogalactan. As our results show, the 20 μg/mL and 30 μg/mL treatment groups were able to maintain the phagocytic ability similarly under the heat stress. However, in the control group without treatment, the phagocytic ability showed a decreasing trend under heat stress.

In our study, we observed that, under treatment with high concentrations of 20 μg/mL and 30 μg/mL, the Annexin-V staining reading data were lower than the control group and 10 μg/mL treatment group. These findings are also similar to the PI-staining data at 12 h. However, at our 24 h observation, the Annexin-V staining and PI-staining data presented no significant difference. We hypothesized that treatment with acetyl-xylogalactan at concentrations of 20 μg/mL and 30 μg/mL only provided a protective ability for 12 h. With long-term exposure to heat stress (41 °C), apoptosis was also presented in the high concentration treatment groups.

Polymorphonuclear cells and peripheral blood mononuclear cells (PBMCs) under heat stress are known to show cellular apoptosis at 41 °C [24]. The intracellular Ca^2+^ level is also related to the cell apoptosis rate via the downregulation of FKBP12.6 expression and the upregulation of phosphorylated-Ser2814-RyR2 and cleaved caspase3 expression [25]. A recent study showed that the mitochondria play a central role in apoptosis, and Ca^2+^ overload is believed to prime the mitochondrial permeability transition pore (mPTP) to open at the stage of myocardial reperfusion, with the intracellular Ca^2+^ levels detected by laser scanning confocal microscopy at 48 h following transfection [26]. In our study, RAW 264.7 treated with acetyl-xylogalactan at 41°C showed reduced cellular apoptosis. However, the *Sarcodia suieae* acetyl-xylogalactan was able to reduce RAW 264.7 macrophage apoptosis only at 12 h under heat stress. To our knowledge, this acetyl-xylogalactan was a large polysaccharide which may not be inserted into the intracellular. As in our previous study, this acetyl-xylogalactan may be interacting with the IL-1 beta receptor on the cell surface to promote downstream signaling [18].

The tumour necrosis factor receptor apoptosis-inducing ligand (TRAIL) and Tarolidine (TRD) have been shown to induce apoptotic cell death and significantly decrease proliferation in HT1080 cells. The expression of several genes has been shown to be related to apoptotic pathways such as *Nfkbia*. TRAIL effectively induced apoptotic cell death in HT1080 fibrosarcoma cells by upregulating *Arhgdia, Tnfaip3* and *Nfkbia* by more than two-fold in comparison with the control [27].

The DNA damage-inducible transcript 3, also known as C/EBP homologous protein (CHOP), is known to be a pro-apoptotic transcription factor*. Ddit3* and putative AP-1 have been putatively identified in the TNFRSF10A promoter, which regulates the extrinsic pathway of apoptosis [28,29]. Animals deficient in both *Jun* and *Ddit3* were evaluated after mechanical optic nerve injury. The combined deficiency of *Jun* and *Ddit3* was more protective of retinal ganglion cell (RGCs) after axonal injury than either *Jun* or *Ddit3* deficiency alone and provided profound long-term protection of RGCs after axonal injury [30]. Research has illustrated that the long-isoform *Cflar* plays a critical role in all three fundamental intracellular processes—autophagy, necroptosis and apoptosis in T lymphocytes [31]—and controls not only the classical death receptor-mediated extrinsic apoptosis pathway but also the nonconventional pattern recognition receptor-dependent apoptotic pathway [32]. To our knowledge, *Cflar*, an inhibitor of apoptosis, regulates apoptosis in vivo, and *Cflar* upregulation was able to induce the resistance of HepG2 cells against Taxol-induced apoptosis [33]. Our results showed that *Sarcodia suieae* acetyl-xylogalactan enhanced *Cflar* gene expression, suggesting that *Sarcodia suieae* acetyl-xylogalactan induced anti-apoptosis factors to protect RAW 264.7 cells against heat-induced apoptosis. Furthermore, we analyzed the RNA sequencing data of RAW 264.7 cells using NGS and proposed a pathway of reduced apoptosis-related genes. As our results confirmed, we detected apoptosis-related genes, such as *Nfkbia, Ddit3, Gadd45a, Akt3* and *Hyou1*.

This study investigated the cellular functions of RAW 264.7 macrophages treated with *S. suieae* acetyl-xylogalactan under heat stress. The predicted expressed genes included *Nfkbia, Hyou1, Ddit3, Gadd45a, Akt3* and *Cflar*). In the RNA-seq analysis, the *S. suieae* acetyl-xylogalactan was demonstrated to have positively regulated apoptosis, phagosome production and IL-17 signaling based on the observed KEGG pathway. For the predicted target genes, *S. suieae* acetyl-xylogalactan facilitated the regulation of the relative apoptotic factors in the DEGseq prediction.

It was already seen in the previous study that mycobacterial HBHA was able to induce endoplasmic reticulum stress-mediated apoptosis through the generation of reactive oxygen species and cytosolic Ca^2+^ in murine macrophage RAW 264.7 cells [34]. It can be seen that while the RAW 264.7 was in the apoptosis situation, the pro-inflammatory cytokines as IL-6 were also produced. Additionally, the evidence demonstrated that the cytokines IL-17A and IL-17F were inducing the RAW 264.7 macrophages autophagy [35].

Our findings show that feeding Nile Tilapia with various concentrations of *S. suieae* acetyl-xylogalactan did not significantly alter the blood’s biochemical parameters. Previous findings illustrated that blood biochemical and immune parameters were considered as important indicator of the fish health [36,37]. Dietary polysaccharides derived from brown macroalgae *Sargassum dentifolium* had no effect on total plasma protein, albumin and globulin levels. Our presented findings also demonstrated a similar condition after feeding with *S. suieae* acetyl-xylogalactan. It was illustrated that feeding the *S. suieae* acetyl-xylogalactan did not affect the Nile Tilapia blood biochemical alternation under heat stress conditions.

Linoleic acid, polyunsaturated fatty acid, has been reported to affect the apoptosis pathway via nuclear transcription factor-kappa B (NF-κB) [38]. Previous study was evidenced that the exogenous arachidonic acid able to induce the apoptosis in colon cancer and other cell lines [39]. It has been shown that arachidonic acid-selective, Ca^2+^-dependent cytosolic phospholipase A2 (cPLA2) is essential for the cytotoxic action of tumor necrosis factor α (TNF-α), leading to the cell apoptosis through the mitochondrial pathway [40]. Arachidonic acid (AA) was also known to induce the activation of NF-κB, cyclooxygenase (COX), lipoxygenase (LOX) and PGE2 formation in cancer and modulate physiological activities as well as tumor progression via metastasis, apoptosis and inflammatory processes [41]. Additionally, the phospholipases A2 (PLA2) reaction is the primary pathway through which arachidonic acid (AA) is released from phospholipids. PLA2s have an important role in cellular death that occurs via necrosis or apoptosis [42]. Combined with our findings, we see that acetyl-xylogalactan reduced the brain arachidonic acid production which is involved in inducing cell and tissue death. Additionally, our findings hypothesized that reduced arachidonic acid downregulated the tissue death through the NF-κB activation.

## 4. Materials and Methods

### 4.1. Sarcodia Suieae Acetyl-Xylogalactan Preparation

The preparation method for *Sarcodia suieae* acetyl-xylogalactan was reported in a previous study [18], and this technique was used in the present study. Briefly, dried *Sarcodia suieae* was treated in water at 60 °C for 6 h. The extracted acetyl-xylogalactan contained galactose (91%) and xylose (9%) as monosaccharides, up to 80.6% polysaccharides and 19.3% acetyl content in the NMR and HPLC analysis. The molecular weight of the acetyl-xylogalactan was 88.5 kDa.

### 4.2. Cellular Findings

#### 4.2.1. Survival of RAW 264.7 Cells under Heat Stress

The mouse macrophage RAW 264.7 cell line was obtained from the Taiwan Bioresource Collection and Research Center (BCRC Number: 60001). Cells were maintained in 90% Dulbecco’s modified Eagle’s medium containing 4 mM L-glutamine, adjusted to contain 1.5 g/L sodium bicarbonate and 4.5 g/L glucose + 10% heat-inactivated fetal bovine serum with 1% Antibiotic Antimycotic Solution (A002, HiMedia Laboratories, India), in a humid field atmosphere of 5% CO_2_ at 37 °C.

To examine the toxicity of *Sarcodia suieae* acetyl-xylogalactan on the RAW 264.7 macrophages, 1 × 10^6^ RAW 264.7 macrophages were treated without (control) and with *Sarcodia suieae* acetyl-xylogalactan at concentrations of 10 µg/mL, 20 µg/mL and 30 µg/mL for 12 and 24 h at 41 °C under 5% CO_2_. At the end of this treatment, a Cell Counting Kit-8 (CCk-8) (B34302, bimake) was used to examine cell survival with a microplate reader at OD 450 nm.

#### 4.2.2. Apoptosis of RAW 264.7 Macrophages under Heat Stress

To examine whether *Sarcodia suieae* acetyl-xylogalactan protected RAW 264.7 macrophages against apoptosis, 1 × 10^6^ RAW 264.7 cells were cultured without (control) and with *Sarcodia suieae* acetyl-xylogalactan at concentrations of 10 µg/mL, 20 µg/mL and 30 μg/mL for 12 h and 24 h at 41 °C under 5% CO_2_. At the end of the treatment period, an Annexin V-FITC Apoptosis Detection kit (AVK250, Strong Biotech Corporation, Taipei city, Taiwan) was used to measure the degree of apoptosis by the fluorescence microplate reader.

#### 4.2.3. Phagocytic Activity of RAW 264.7 Cells under Heat Stress

The phagocytic activity of RAW 264.7 cells was determined by the EZ CellTM Phagocytosis Assay kit (Green *E. coli*). Briefly, 1 × 10^6^ RAW 264.7 cells were cultured without (control) and with 10 µg/mL, 20 µg/mL and 30 μg/mL of *Sarcodia suieae* acetyl-xylogalactan and incubated at 41 °C under 5% CO_2_ for 12 and 24 h. Subsequently, the culture medium was removed and phagocytic activity was detected at Ex/Em = 490/520.

#### 4.2.4. Observation of Galactosidase Expression in RAW 264.7 Cells under Heat Stress

The expression of galactosidase in RAW 264.7 cells was monitored by the Senescent Detection Kit (Cat. K320-250, Biovision, Waltham, MA, USA) under a microscope. Briefly, 1 × 10^6^ RAW 264.7 cells were cultured without (control) and with 10 µg/mL, 20 and 30 µg/mL of *Sarcodia suieae* acetyl-xylogalactan and incubated at 41 °C under 5% CO_2_ for 12 h. Subsequently, the culture medium was removed and observed under the microscope.

#### 4.2.5. Mitochondrial Membrane Potential Changes in RAW 264.7 Cells after Heat Stress

To analyze mitochondrial membrane potential changes in RAW 264.7 cells, 1 × 10^6^ RAW 264.7 cells were pre-cultured without (control) and with 10 μg/mL, 20 μg/mL and 30 μg/mL *Sarcodia suieae* acetyl-xylogalactan for 24 h at 37 °C under 5% CO_2_. The end of the pre-treatment, the experiment RAW 264.7 cells were incubated for 24 h at 41 °C under 5% CO_2_. At the end of the incubation period, the culture medium was removed, and the mitochondrial membrane potential changes were determined for 24 h using JC-9 Dye (Mitochondrial Membrane Potential Probe) (D22421, Thermo, Waltham, MA, USA) to determine the green fluorescence changes in the mitochondrial membrane potential after heat stress. Green fluorescence was detected at an excitation of 535 nm and emission of 590 nm using the Spectra max Gemini XPS (Molecular Device).

#### 4.2.6. Intracellular Ca^2+^ Concentration in RAW 264.7 Cells after Heat Stress

For analysis of intracellular Ca^2+^ expression, the control and experimental groups were formed as in the previous assessments. An amount of 1 × 10^6^ RAW 264.7 cells were pre-cultured without (control) and with 10 μg/mL, 20 μg/mL and 30 μg/mL *Sarcodia suieae* acetyl-xylogalactan for 24 h at 37 °C under 5% CO_2_. At the end of the pre-treatment, the experiment RAW 264.7 cells were incubated for 24 h at 41 °C under 5% CO_2_. At the end of the incubation period, the culture medium was removed. At the end of the incubation period, the culture medium was removed, and intracellular Ca^2+^ levels were continually determined for 24 h using the Fluo-4 Direct Calcium Assay Kit (F10471; Thermo, Waltham, MA, USA).

#### 4.2.7. Gene Expression

##### RNA Sequencing (Transcriptome)

The control and experimental groups were formed as in the previous assessments and incubated at 41 °C under 5% CO_2_ for 24 h. RAW 264.7 macrophages (1 × 10^6^ cells) were treated without (control) and with 10 μg/mL, 20 μg/mL and 30 μg/mL *S. suieae* acetyl-xylogalactan for 24 h at 41 °C under 5% CO_2_. Thereafter, RNA was isolated from the cells using Azol RNA Isolation Reagent (Arrowtech, New Taipei City, Taiwan). RNA concentrations were determined using Nanodrop. Then, 1 µg of RNA was sent to Biotools Co., Ltd., Taiwan, for the RNA sequencing analysis, where NovaSeq (San Diego, CA 92122 USA). 6000 Sequencing System (Illumina, San Diego, CA, USA) was used.

The reference genome was mapped using HISAT2. Only filtered reads could be used to analyze the mapping status of RNA-seq data to the reference genome. The original data, obtained by high-throughput sequencing (Illumina NovaSeq 6000 platform, San Diego, CA 92122 USA), were transformed into raw sequenced reads by CASAVA base calling and stored in FASTQ format. FastQC and MultiQC were used to check fastq files for quality. The obtained raw paired-end reads were filtered by Trimmomatic (v0.38) (Germany.) to discard low-quality reads, trim adaptor sequences and eliminate poor-quality bases with the following parameters: LEADING:3 TRAILING:3 SLIDINGWINDOW:4:15 MINLEN:30. The obtained high-quality data (clean reads) were used for subsequent analysis. Read pairs from each sample were aligned to the reference genome (e.g., *H. sapiens*, GRCh38) using the HISAT2 software (v2.1.0). Feature Counts (v1.6.0) was used to count the read numbers mapped to individual genes. For gene expression, the “Trimmed Mean of Mvalues” normalization (TMM) was performed using DEGseq (v1.36.1) without biological duplicates, and the “Relative Log Expression” normalization (RLE) was performed using DESeq2 (v1.22.1) with biological duplicates. The differentially expressed genes (DEGs) analysis of two conditions was performed in R using DEGseq (without biological replicates) and DESeq2 (with biological replicates), based on negative binomial distribution and Poisson distribution model, respectively. The resulting *p*-values were adjusted using Benjamini and Hochberg’s approach for controlling the FDR. The GO and KEGG pathway enrichment analysis of DEGs were conducted using cluster Profiler (v3.10.1). The DOSE package was used to map the disease ontology (DO) terms to MeSH, ICD, NCI’s thesaurus, SNOMED and OMIM. Gene set enrichment analysis (GSEA) was performed with 1000 permutations to identify enriched biological functions and activated pathways from the molecular signatures database (MsigDB). MsigDB is a collection of annotated gene sets for use with GSEA software, including hallmark gene sets, positional gene sets, curated gene sets, motif gene sets, computational gene sets, GO gene sets, oncogenic gene sets and immunologic gene sets. The protein–protein interaction (PPI) network was constructed for differential expression genes using STRINGdb (https://string-db.org/). In addition, weighted gene co-expression network analysis (WGCNA) was constructed with the co-expression network based on the correlation coefficient of the expression pattern using the WGCNA (v1.64) package.

#### 4.2.8. Real-Time qPCR Analysis

The real-time qPCR analysis of the macrophages *Nfkbia, Ddit3, Gadd45a, Akt3, Hyou*1 and *Cflar* was performed by Biotools Co., Ltd. The quantitative real-time PCR service was performed using the TOOLS 2xSYBR qPCR Mix Kit (FPT-BB05). The TOOLS 2xSYBR qPCR Mix Kit is specially designed to perform real-time PCR in SYBR Green I fluorescent-based detection assays. TOOLS 2xSYBR qPCR Mix adopts a unique dual hot-start enzymes system (chemically modified HotStar Taq DNA polymerase and antibody-modified Anti Taq DNA Polyerase). In combination with the preoptimized buffer solution, TOOLS 2xSYBR qPCR Mix provides a convenient format for highly sensitive and specific qPCR amplification.

The qPCR protocol was as below:

1. 95.0 °C for 15:00;
2. 95.0 °C for 0:10;
3. 62.0 °C for 0:30, Plate Read;
4. GOTO 2, 59 more times;
5. 95.0 °C for 0:10;
6. Melt Curve 65.0 to 95.0 °C, Increments of 0.5 °C every 0:05;
7. Plate Read.

Beta-actin was used as the reference in the comparative CT to determine the relative alteration. Fluorescence was analyzed using the auto CT method to determine the threshold of each gene, and the 2^−ΔΔCT^ method was used to calculate CT values using StepOne (version 2.3). Data are presented as fold changes in the mRNA level normalized to the reference gene β-actin. The following oligonucleotide sequences were used for creating qPCR primers.
**Gene****Forward primer****Reverse Primer***β-actin*CATTGCTGACAGGATGCAGAAGGTGCTGGAAGGTGGACAGTGAGG*Nfkbia*GGTGACTTTGGGTGCTGAT CTTGGTAGGTTACCCTGTTGAC*Ddit3*TCCTGTCCTCAGATGAAATTGGGCAGGGTCAAGAGTAGTGAAG*Gadd45a*CTGTGTGCTGGTGACGAA GCACCCACTGATCCATGTAG*Akt3* CAGAACGACCAAAGCCAAATACCTTCCGTCCACTCTTCTCTTTC*Hyou1*CGCAAAGTCATCACCTTTAACCGTCAGATTCTGGGAGCCAAATA*Cflar* CTGATTATAGGGTCCTGCTGATGTTGCCTCTGCCTGTGTAATC

#### 4.2.9. Cytokines of IL-6 and IL-17A Production

IL-6 and IL-17A production in the RAW 264.7 macrophages treated with or without *S. suieae* acetyl-xylogalactan was analysed under heat stress. In brief, 1 × 10^6^ RAW 264.7 macrophages were cultured in a 96-well plate with or without 10, 20, or 30 μg/mL *S. suieae* acetyl-xylogalactan under 37 and 41 °C for 24 h. The culture medium was then analysed using the ELISA IL-6 and IL-17A assay kit (QIAGEM, SEM03015A and SEM03023A).

### 4.3. In Vivo Experiment

Male Tilapias (*Oreochomis niloticus*) fish were obtained from the Sheng-Diao Aquatic Technology, Taiwan. After transferring to the laboratory, Tilapias were cultured until the body weight of each Tilapia approximately 65 g. This experiment was used five individuals per group and replicated it three times. Under heat stress, the experiment Tilapia was cultured at 25 °C (control) and 35 °C. This experiment was following the animal procedure was following the Guide for Animal Use Protocol of the Institutional Animal Care and Use Committee (IACUC) of National Pingtung University of Science and Technology. The IACUC Approval No. was NPUST-108-071.

The diet was prepared according to the commercial Tilapia feed (SHYE YIH FEEDING CO., LTD). Dried acetyl-xylogalactan was dissolved in sterile D.D. water and sprayed on the commercial feed. The food was gently mixed at room temperature (25 °C) and baked at 60 °C for complete drying.

Experimental feed containing 0 g/Kg-Feed (Control), 10 g/Kg-Feed, 20 g/Kg-Feed or 30 g/Kg-Feed acetyl-xylogalactan was used to feed Nile Tilapia two times daily; the daily feed intake of Nile tilapia was equal to 5% of their weight at 8:00 and 16:00. The siphon cleaning of the tank bottom was conducted every 2 days.

At 25 °C (control), the experiment Tilapia was feeding with 0 g/Kg-Feed (control). At 35°C (experiment), the experiment Tilapia was divided into feeding with 0 g/Kg-Feed (Control), 10 g/Kg-Feed, 20 g/Kg-Feed or 30 g/Kg-Feed acetyl-xylogalactan. The feeding experiment was performed for 4 weeks. At the end of the experiment, Tilapias were sacrificed and the brain was for LC-MS metabolites analysis, liver was obtained for the histopathology, and peripheral blood were collected for the biochemical analysis.

#### 4.3.1. Blood Biochemical and IL-10 Analysis

Five fishes were obtained from each experimental tank at the end of the treatment. Blood samples (1 mL) were collected from the caudal vessels using heparinised syringes and placed in sterile tubes for biochemical analysis. Any haemolysed, clotted or insufficient samples were discarded. Plasma samples were analysed for glucose, total cholesterol, blood urea nitrogen (BUN), total bilirubin, aspartate aminotransferase, alanine aminotransferase, total protein, albumin, calcium and uric acid (UA) using an automated blood biochemical analyser (SPOTCHEM EZ SP-4430, ARKRAY). Fish Interleukin 10 ELISA Kit was purchased form MyBiosourse, San Diego, USA, Cat. No MBS1601741.

#### 4.3.2. LC-MS Metabolite Analysis

The end of the feeding experiment, the control and experiment fish was sacrificed and collected the brain. Five independent fish brain was collected for each group. After collect, the sample was transferred to the analysis company, BIOTOOLS Co., Ltd. (Taiwan) for the LC-MS metabolites exchange.

#### 4.3.3. Histological Analysis and Hepatic Glycogen Observation

Liver was fixed with 10% formalin. Paraffin-embedded liver tissues were cut at 5 µm thickness and stained by hematoxylin–eosin (H&E), Periodic Acid–Schiff stain (PAS) and Giemsa stain for microscopy.

### 4.4. Statistical Analysis

One way ANOVA was carried out with Scheffe’s post hoc test was used in this significance difference analysis. A *p*-value less than 0.05 was considered statistically significant. The results were presented as means ± SD (* *p* < 0.05 and ** *p* < 0.001).

The RNA-seq data were obtained as differences between the treatment and control groups. DEGseq was used to analyze the statistical significance between the treatment and control groups. Relative log expression was higher than 2, and the corrected *p*-value was less than 0.005, which was considered to indicate statistical significance.

The LC-MS raw data were converted to the mzXML format using Proteo Wizard and processed with an in-house program, which was developed using R and based on XCMS, for peak detection, extraction, alignment and integration. An in-house MS2 database (Biotree DB) was then applied in metabolite annotation. The cut-off for annotation was set at 0.5.

## 5. Conclusions

In conclusion, these findings show that *Sarcodia suieae* acetyl-xylogalactan was able to induce anti-apoptotic *cflar* gene expression and downregulate the expression of the apoptosis factors *Ddit3* and *Hyou1*. Combined with the Nile Tilapia findings, it is known to reduce brain arachidonic acid expression, which has been proposed to be involved in tissue death via downstream NF-κB activation.

## 6. Patents

There is no patent resulting from the work reported in this manuscript.

## Figures and Tables

**Table 1:** The Nile Tilapia blood biochemical analysis. Serum indices of Nile Tilapia fed with acetyl-xylogalactan for 4 weeks under heat stress, * indicates *p* < 0.05, wherein each treatment group was compared to the control group.

**Item**	**Glu**	**T-Cho**	**BUN**	**T-Bil**	**GOT**	**GPT**	**T-Pro**	**Alb**
**Unit**	**mg/dL**	**mg/dL**	**mg/dL**	**mg/dL**	**IU/L**	**IU/L**	**g/dL**	**g/dL**
25 °C	318 ± 46.81	115 ± 4.16	5 ± 0	13.5 ± 0.10	56 ± 14.19	22 ± 11.14	10.87 ± 0.23	3.02 ± 0.26
35 °C—0 g/Kg-Feed	333 ± 8.33	120 ± 2.00	5 ± 0	14.07 ± 0.15	51 ± 11.53	11 ± 1.15	9.67 ± 2.31	2.87 ± 0.76
35 °C—10 g/Kg-Feed	381 ± 7.81	127 ± 10.07	5 ± 0	15.23 ± 0.38 *	95 ± 11.50	28 ± 12.77	11 ± 0.00	3.27 ± 0.21
35 °C—20 g/Kg-Feed	279 ± 57.36	132 ± 19.35	5 ± 0	13.13 ± 1.10	101 ± 34.43	30 ± 7.51	9.8 ± 1.51	2.93 ± 0.55
35 °C—30 g/Kg-Feed	324 ± 28.10	124 ± 14.93	5 ± 0	13.8 ± 0.10	47 ± 4.93	13 ± 3.00	10.87 ± 0.23	3.27 ± 0.15
**Item**	**Ca**	**TG**	**UA**	**LDH**	**Alb/T-Pro**	**CPK**	**ALP**	**Mg**
**Unit**	**mg/dL**	**mg/dL**	**mg/dL**	**IU/L**	**Ratio**	**IU/L**	**IU/L**	**mg/dL**
25 ℃	20 ± 0.00	112 ± 61.61	20 ± 0.00	4000 ± 0.00	0.29 ± 0.02	2000 ± 0	79.67 ± 11.55	3.13 ± 0.15
35 °C—0 g/Kg-Feed	20 ± 0.00	70 ± 21.08	20 ± 0.00	4000 ± 0.00	0.30 ± 0.01	2000 ± 0	86.33 ± 16.86	3.30 ± 0
35 °C—10 g/Kg-Feed	20 ± 0.00	75 ± 10.82	20 ± 0.00	4000 ± 0.00	0.30 ± 0.02	1765 ± 407.03	91 ± 21.79	3.67 ± 0.51
35 °C—20 g/Kg-Feed	20 ± 0.69	122 ± 7.57	20 ± 0.00	3169 ± 1439.33	0.30 ± 0.01	2000 ± 0	68 ± 16.70	3.47 ± 0.21
35 °C—30 g/Kg-Feed	20 ± 0.00	106 ± 42.93	20 ± 0.00	4000 ± 0.00	0.30 ± 0.66	1993 ± 10.97	113 ± 35.93	3.73 ± 0.49
**Item**	**HDL-c**	**Amy**	**IP**	**GGT**	**Cre**	**FRA**	**IL-10**	
**Unit**	**mg/dL**	**IU/L**	**mg/dL**	**IU/L**	**mg/dL**	**umol/L**	**ng/L**	
25 °C	108 ± 8.89	18.33 ± 0.58	16.5 ± 0.53	10.67 ± 1.15	0.3 ± 0	88 ± 4	ND.	
35 °C—0 g/Kg-Feed	129 ± 8.08	10 ± 0 *	16.1 ± 0.82	10 ± 0	0.3 ± 0	84 ± 7.51	ND.	
35 °C—10 g/Kg-Feed	100 ± 17.90	10 ± 0 *	19.3 ± 1.21	10 ± 0	0.33 ± 0.06	87 ± 3.61	ND.	
35 °C—20 g/Kg-Feed	116 ± 9.29	16.33 ± 3.79	14.33 ± 2.12	10 ± 0	0.3 ± 0	87 ± 4.93	ND.	
35 °C—30 g/Kg-Feed	102 ± 8.96	12.33 ± 4.04	17.27 ± 3.37	12 ± 1.73	0.3 ± 0	91 ± 8.50	ND.	

**Table 2:** Effect of the Nile Tilapia metabolites exchange. The brain of Nile tilapia is fed with acetyl-xylogalactan for 4 weeks under heat stress. The red mark denotes downregulation compared to the 25 °C (control). The green mark was meant to up regulation compared to the 25 °C (control).

**35 °C—0 g/Kg-Feed vs. 25 °C**					
**featureID**	**CpdName**	**Score**	**KEGGID**	**pvalue**	**log2foldchange**
FT0730	Ginkgolide A	0.61611		0.027372015	−1.290974201
FT0213	2-Hydroxyethanesulfonate	0.99537	C05123	0.015537504	−0.799873206
FT0013	Phosphoenolpyruvic acid	0.99915	C00074	0.012554802	1.155737014
**35 °C—10 g/Kg-Feed vs. 25 °C**					
**featureID**	**CpdName**	**Score**	**KEGGID**	**pvalue**	**log2foldchange**
FT0213	2-Hydroxyethanesulfonate	0.99537	C05123	0.02122731	−0.552585532
FT0730	Ginkgolide A	0.61611		0.031505868	−1.258174039
**35 °C—20 g/Kg-Feed vs. 25 °C**					
**featureID**	**CpdName**	**Score**	**KEGGID**	**pvalue**	**log2foldchange**
FT0730	Ginkgolide A	0.61611		0.012228984	−2.056046835
FT1562	Arachidonic acid	0.99801	C00219	0.017941797	−0.883800718
**35 °C—30 g/Kg-Feed vs. 25 °C**					
**featureID**	**CpdName**	**Score**	**KEGGID**	**pvalue**	**log2foldchange**
FT0507	S-Lactoylglutathione	0.70373	C03451	0.043343983	−0.754247254
FT1562	Arachidonic acid	0.99801	C00219	0.039958685	−0.950275021
FT0730	Ginkgolide A	0.61611		0.015836172	−2.033361388

## Data Availability

The datasets generated and analyzed during the current study are available in the NCBI repository with the BioProject database at http://www.ncbi.nlm.nih.gov/bioproject/686826 (accessed 21 December 2020).
